# Prognostic impact of blood and urinary angiogenic factor levels at diagnosis and during treatment in patients with osteosarcoma: a prospective study

**DOI:** 10.1186/s12885-017-3409-z

**Published:** 2017-06-15

**Authors:** Marie-Dominique Tabone, Laurence Brugières, Sophie Piperno-Neumann, Marie-Ange Selva, Perrine Marec-Bérard, Hélène Pacquement, Cyril Lervat, Nadège Corradini, Jean-Claude Gentet, Rémy Couderc, Aurélie Chevance, Céline Mahier-Ait Oukhatar, Natacha Entz-Werle, Jean-Yves Blay, Marie-Cecile Le Deley

**Affiliations:** 1 Department of Paediatric Onco-Haematology, Armand Trousseau Hospital, 26 avenue du Dr A. Netter, 75571 Paris cedex12, France; 2 Department of Children and adolescents Oncology, Gustave Roussy Cancer campus, 114 rue E. Vaillant, 94805 Villejuif, France; 3 Department of Medical Oncology, Curie Institute, 26 rue d’Ulm, 75005 Paris, France; 4 Department of Biochemistry, Armand Trousseau Hospital, 26 avenue du Dr A. Netter, 75571 Paris cedex12, France; 5 Institute for Paediatric Haematology and Oncology, Léon Bérard Cancer Centre, 28 rue Laennec, 69008 Lyon, France; 6 Department of Paediatric Oncology, Curie Institute, 26 rue d’Ulm, 75005 Paris, France; 7 Department of Paediatric Oncology, Oscar Lambret Cancer Centre, 3 rue Frédéric Combemale, 59020 Lille cedex, France; 8 Department of Paediatric Haemato-Oncology, Mother and Child Hospital, 7 quai Moncousu, 44093 Nantes, France; 9 Department of Paediatric Oncology, La Timone Hospital, 264 rue Saint Pierre, 13385 Marseille cedex 5, France; 10 Biostatistics and Epidemiology unit, Gustave Roussy Cancer Campus, 114 rue E. Vaillant, 94805, Villejuif, France; 11 Department of Clinical Studies, Unicancer, 101 rue de Tolbiac, 75654, 13 Paris cedex, France; 12 Department of Paediatric Oncology, Hautepierre Hospital, 1 avenue Molière, 67100 Strasbourg, France; 13 Department of Medical Oncology, Léon Bérard Cancer Centre, 28 rue Laennec, 69008 Lyon, France; 14 Claude Bernard University Lyon 1, 43 boulevard du 11 novembre 1918, 69622 Villeurbanne cedex, France; 15 Paris-Saclay University, Univ. Paris-Sud, CESP, INSERM, Gustave Roussy, Villejuif, France; 16 Department of Paediatric Onco-Haematology AP-HP, GHUEP, Trousseau La Roche-Guyon Hospital, 26 avenue du Dr A. Netter, 75012 Paris, France

**Keywords:** Osteosarcoma, Angiogenic factors, Vascular endothelial growth factor, Basic fibroblast growth factor

## Abstract

**Background:**

Angiogenesis is essential for the progression and metastatic spread of solid tumours. Expression of vascular endothelial growth factor (VEGF) has been linked to poor survival among osteosarcoma patients but the clinical relevance of monitoring blood and urine angiogenic factors is uncertain. The aim of this study was to determine the prognostic significance of blood VEGF and blood and urinary basic fibroblast growth factor (bFGF) levels in osteosarcoma patients, both at diagnosis and during treatment.

**Methods:**

Patients with localised or metastatic osteosarcoma enrolled in OS2005 and OS2006 studies between 2005 and 2011 were prospectively included in this study. VEGF and bFGF levels in serum and plasma and bFGF levels in urine were measured by ELISA at diagnosis, before surgery, and at the end of treatment. Endpoints considered for the prognostic analysis were histological response, progression-free and overall survival. Kruskal-Wallis tests were used to compare the distribution of baseline biomarker values across the different subgroups, and paired sample Wilcoxon rank tests were used to analyze changes over time. Association between biomarker levels and outcomes were assessed in multivariable models (logistic regression for histologic response, and Cox models for survival).

**Results:**

Samples were available at diagnosis for 269 patients (54% males; age ≤ 18 years: 73%; localised disease in 68%, doubtful lung lesions in 17%, and metastases in 15%). High serum VEGF and bFGF levels were observed in respectively 61% and 51% of patients. Serum and plasma VEGF values were not strongly correlated with one another (*r* = 0.53). High serum and plasma VEGF levels were significantly more frequent in patients with large tumours (≥10 cm; *p* = 0.003 and *p* = 0.02, respectively). VEGF levels fell significantly during pre-operative chemotherapy (*p* < 0.0001). No significant correlation was found between this variation and either the histological response, progression-free survival or overall survival (*p* = 0.26, *p* = 0.67, and *p* = 0.87, respectively). No significant association was found between blood or urinary bFGF levels and clinical characteristics, histological response, or survival.

**Conclusions:**

Levels of VEGF and bFGF angiogenic factors are high in most osteosarcoma patients, but have no significant impact on response to chemotherapy or outcome in this large prospective series.

**OS 2006 trial registration number:**

clinicaltrials.gov NCT00470223; date of registration: May 3th 2007.

**Electronic supplementary material:**

The online version of this article (doi:10.1186/s12885-017-3409-z) contains supplementary material, which is available to authorized users.

## Background

Osteosarcoma is the most common malignant bone tumour in adolescents and young adults. Despite considerable improvements in survival with chemotherapy, patients with metastases at diagnosis and patients who relapse still have a poor prognosis [1, 2]. New therapeutic approaches are needed for those patients.

Angiogenesis is essential for the growth, progression and metastatic spread of solid tumours [3]. Tumour adoption of an angiogenic phenotype is believed to involve a change in the balance between angiogenic inducers and inhibitors. Several studies have suggested that microvessel density and vascular endothelial growth factor (VEGF) expression in untreated osteosarcoma patients are associated with pulmonary metastasis and poor survival [4–8], but conflicting results have been reported [9, 10]. Most of these studies were based on immunohistochemical methods, which are difficult to standardize. Serum assays are more reproducible and allow repeated measurements over time. Among the different angiogenic factors, elevated levels of bFGF (basic fibroblast growth factor) and VEGF have been detected in serum and/or urine of adults and children with malignancies, including osteosarcoma [11–14]. However, a better knowledge of angiogenic factor levels and kinetics in body fluids during treatment is needed. The aim of this study was to determine blood VEGF levels, and blood and urinary bFGF levels in osteosarcoma patients, and to investigate whether values at diagnosis or changes during treatment are associated with disease characteristics or outcome.

## Methods

### Patients

Samples were collected in two consecutive cohorts of French newly diagnosed high-grade osteosarcoma patients included between January 2005 and December 2011 in OS2005 study aiming to collect samples for biological research in patients treated with standard chemotherapy before the opening of OS2006 trial (NCT00470223), a national study including a randomised trial evaluating zoledronate and collection of samples for biological research. Written informed consent was obtained from patients and/or their parents/guardians. Patients included in OS2005 study were aged below 25 and received preoperative chemotherapy based on high-dose methotrexate (HDMTX) plus etoposide-ifosfamide [15]. In OS2006 trial, patients under 18 years received the same HDMTX-based chemotherapy as in OS2005 study; patients over 25 years old received doxorubicin, ifosfamide and platinum [16]; and patients between 18 and 25 received either HDMTX-based chemotherapy or the adult regimen, as decided by each participating centre at the beginning of the study. Post-operative treatment was adapted to the histological response. In trial OS2006, patients could be randomized to receive zoledronate or not in addition to chemotherapy [17].

### Angiogenic factor assays

VEGF levels in serum and plasma, and bFGF (also called FGF2) levels in serum, plasma and urine were evaluated at three time points: at osteosarcoma diagnosis (T0: at diagnosis), after preoperative chemotherapy (T1: before surgery of the primary tumour), and at the end of treatment (T2). Samples were collected in dry sterile tubes (serum and urine) or EDTA tubes (plasma) and immediately stored in aliquots at −80 °C. The frozen samples were sent to a central biochemistry laboratory for analysis. Isoform VEGF-165 (serum and plasma) and bFGF (serum, plasma and urine) levels were assayed as previously described [18], with sandwich enzyme linked immunoassay methods (Quantikine; R & D systems, Minneapolis, MN). Each sample was tested in duplicate. The bFGF concentration in urine was expressed in nanograms per gram of creatinine.

### Statistical considerations

The distribution of each biomarker was described using standard statistics. A high value was defined as a value higher than the published cutoff obtained with the same sandwich enzyme immunoassay method (upper limit of the 95% confidence interval of the mean value in healthy controls) [14, 18], considering separately children (<18 years) and adults (≥18 years). Correlations between the different biomarkers were tested using Spearman’s rank correlation coefficient.

For each biomarker, Kruskal-Wallis non parametric tests were used to compare the distribution of values at diagnosis across the different patient subgroups, in terms of gender, age (<13 versus 13–18 versus >18 years), tumour size (<10 cm versus ≥10 cm), the histological subtype, disease stage at diagnosis (localised, metastatic disease, doubtful lesions) and the alkaline phosphatase level at diagnosis (<1.25 versus >1.25 times the upper limit of normal).

For each biomarker, paired sample Wilcoxon signed rank tests were used to analyze changes over time (T1-T0 and T2-T1).

Three endpoints were considered for the prognostic analysis: the histological response to pre-operative chemotherapy, the progression-free survival rate and the overall survival rate.

A poor histological response to pre-operative chemotherapy was defined as a mean percentage of viable cells >10%. For each biomarker, the distribution of baseline values was compared between patients with good and poor histological responses, using the Kruskal-Wallis test. A similar approach was used to analyse biomarker variations during pre-operative chemotherapy. The influence of baseline values and changes over time on the risk of a poor histological response was modelled by multivariable logistic regression. In order to explore the shape of the possible relationship, we considered the quartiles of the distribution of each biomarker. An additional analysis was adjusted on the treatment group (with or without zoledronate).

Progression-free and overall survival curves were constructed with the Kaplan-Meier method. Progression-free survival (PFS) was defined as the time from initial biopsy to treatment failure (progression, relapse, or death of any cause). Overall survival (OS) was measured from the time between biopsy and death, whatever the cause. We examined the influence of biomarker values at diagnosis, changes during pre-operative chemotherapy adjusted for the value at diagnosis, and the values at the end of treatment. The association of each biomarker with the survival outcomes was evaluated by using Cox regression models stratified by treatment group (with or without zoledronate) and the disease stage at diagnosis. A sensitivity analysis was restricted to patients with high biomarker levels at diagnosis.

To take into account multiple comparisons in the prognostic analysis, we set the *P* value for significance at 8 × 10^−4^ using the Bonferroni correction. All statistical analyses used SAS software v9.3 (SAS Institute, Cary, NC).

### Results

### Patient characteristics

Among the 456 patients included in the OS2005 study or OS2006 trial between January 2005 and December 2011, 269 patients had at least one available sample at diagnosis (28 in OS2005 and 241 in OS2006). They were recruited in 40 French paediatric or adult oncology departments from Société Française de Lutte contre les Cancers et Leucémies de l’Enfant et de l’Adolescent (SFCE) and Groupe Sarcome Français - Groupe d’Étude des Tumeurs Osseuses (GSF-GETO). The participant flow chart is shown in Additional file 1: Fig. S1. Patient characteristics are described in Table 1. Median age was 15.0 years (range, 1.4–50.4). Patients evaluated for angiogenic factors at diagnosis (study patients) had similar initial characteristics to the remaining patients (see Additional file 2: Table S1).

**Table 1:** Serum-VEGF, serum-bFGF and urinary-bFGF levels at diagnosis, according to patient and tumour characteristics

	Serum VEGF (pg/mL)			Serum bFGF (pg/mL)			Urinary bFGF (ng/g creatinine)		
	*N*	Median [Q1-Q3]^a^	*P value* ^b^	*N*	Median [Q1-Q3]^a^	*P value* ^b^	*N*	Median [Q1-Q3]^a^	*P value* ^b^
All	246	428 [274–685]		242	4.3 [3–13]		129	5.2 [2.7–10.4]	
Gender			0.94			0.10			0.30
Male	136	432 [286–675]		133	5 [3–14]		70	4.5 [2.4–9.6]	
Female	110	427 [252–686]		109	4 [3–11]		59	5.6 [3.1–10.9]	
Age			0.65			0.07			0.03
< 13 years	71	466 [223–692]		71	7 [3–18]		36	7.5 [4.8–12.9]	
13–18 years	106	399 [271–686]		104	4 [3–13]		59	4.1 [2.2–8.9]	
> 18 years	69	440 [327–642]		67	3 [3–10]		34	5.3 [1.9–11.4]	
Tumour size			0.003			0.24			0.28
< 10 cm	110	391 [222–567]		108	5 [3–12]		59	4.9 [2.1–9.6]	
≥ 10 cm	121	504 [308–738]		119	3 [3–13]		62	5.3 [2.9–11.6]	
Initial stage ^c^			0.64			0.16			0.17
Localized	164	431 [258–689]		162	4 [3–11]		92	5176 [2.4–9.2]	
Doubtful lesions	40	418 [335–711]		39	7 [3–15]		21	4.9 [3.3–12.2]	
Metastases	39	396 [274–621]		38	3 [3–7]		15	9.6 [3.5–17.1]	
Histologic subtype			0.36			0.57			0.46
Osteoblastic	154	435 [303–735]		152	5 [3–13]		82	5.3 [2.8–10.8]	
Fibroblastic	10	399 [205–512]		11	7 [3–13]		2	2.6 [1.8–3.3]	
Chondroblastic	40	461 [280–610]		38	4 [3–11]		26	5.5 [3.5–11.4]	
Telangiectasic	7	283 [178–692]		7	3 [3–3]		4	5.3 [4–77.3]	
Other	25	365 [241–484]		24	4 [3–14]		13	3.1 [1.8–7.9]	
Alkaline phosphatase			0.11			0.21			0.33
< 1.25 x ULN	137	404 [252–644]		135	5 [3–11]		74	4.9 [2.5–9.6]	
≥ 1.25 x ULN	70	472 [305–736]		69	3 [3–13]		40	6.1 [3–13.6]	
Histological response ^d^			0.92			0.40			0.44
Good	138	435 [283–732]		137	5 [3–13]		85	5.1 [2.9–10.2]	
Poor	76	429 [314–647]		74	4 [3–11]		31	4.1 [2.1–8.9]	

^a^[Q1-Q3]: inter-quartile range

^b^Kruskal-Wallis test comparing the distributions between the different subsets

^c^Lung metastases on CT were defined by ≥1 nodule >10 mm, and/or ≥2 nodules from 5 to 9 mm, and/or ≥5 well limited nodules <5 mm. Other types of lesion were considered as doubtful lesions

^d^Results by quartile of the distributions are available in Additional file 5 Table-S3

With a median follow-up of 3.3 years, 96 treatment failures occurred, consisting of 95 relapses or progressions and one treatment-related death. A total of 48 patients died, all but two from disease progression.

### Biomarker levels at diagnosis and changes over time

**Fig. 1:**
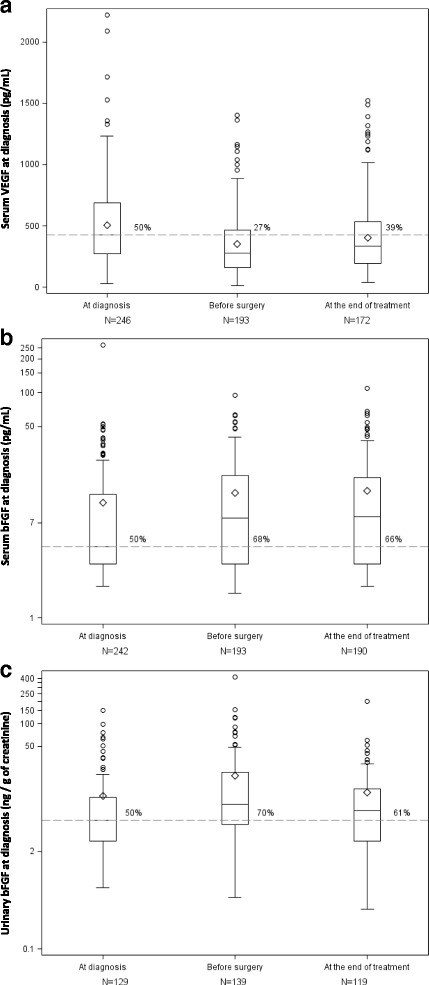
Variations in biomarkers over time. Panel A: serum VEGF, Panel B: serum bFGF, and Panel C: urinary bFGF.  Median angiogenic factor levels at diagnosis (428 pg/mL for serum VEGF,4.3 pg/mL for serum bFGF and 5.2 ng/g of creatinine for urinary bFGF). At each time point, the percentage given above the dashed line corresponds to the percentage of cases with a value above the median value at diagnosis. The bottom and top edges of the box indicate the inter-quartile range (Q1-Q3, IQR). The line inside the box indicates the median value, and the small diamond indicates the mean. The whiskers indicate the minimum value (respectively, maximum) higher (respectively, lower) than Q1–1.5*IQR (respectively, Q3 + 1.5*IQR). The results were similar when only patients with results available at the three time points were analyzed

The distribution of serum VEGF and serum and urinary bFGF levels at diagnosis is shown in Table 1 and illustrated in Fig. 1. Respectively 149/246 (61%), 123/242 (51%) and 124/129 (96%) patients had high serum VEGF, serum bFGF and urine bFGF values at diagnosis. Distribution of plasma VEGF and bFGF levels at diagnosis is shown in Additional file 3: Table S2.

The serum VEGF level at diagnosis was significantly associated with tumour size (median 504 and 391 pg/mL for tumours ≥ and <10 cm, respectively; *p =* 0.003), but not with other patient or tumour characteristics. We found no significant association between bFGF levels at diagnosis and other baseline characteristics, apart from a relation between age and urinary bFGF levels, which were higher in patients younger than 13 years than in older patients (*p =* 0.03).

As illustrated in Additional file 4: Fig. S2, serum and plasma levels were not strongly correlated with one another (correlation coefficient 0.53 for VEGF, 0.22 for bFGF). The correlation between VEGF and bFGF levels was also weak (0.18 for serum, 0.34 for plasma).

As illustrated in Fig. 1, serum VEGF levels fell significantly between baseline (T0) and the pre-surgical (T1) assessment (median − 79 pg/mL, *p* < 0.0001), and rose slightly between T1 and T2 at the end of treatment (median + 43 pg/mL, *p* = 0.0006). The decline in serum VEGF levels during pre-operative chemotherapy was unaffected by the use of zoledronate (*p* = 0.98). Serum bFGF levels increased slightly from T0 to T1 (median + 0.84 pg/mL, *p* = 0.01), then remained stable (median = 0.0, *p* = 0.92). We also observed a significant change in urinary bFGF levels over time, with an initial increase during pre-operative chemotherapy (median + 2.6 ng/g of creatinine, *p* = 0.001) followed by a significant decrease (median − 3.6 ng/g of creatinine, *p* = 0.02).

### Impact of biomarker levels and kinetics on patient outcomes

We observed no significant association between angiogenic factor levels at diagnosis and the risk of a poor histological response to chemotherapy in univariate analysis (Kruskal-Wallis tests for comparison of distributions: *p* = 0.92, *p* = 0.40 and *p* = 0.44 respectively for serum VEGF, serum bFGF and urinary bFGF, Table 1). The absence of significant association was confirmed in multivariable analysis (*p* = 0.44, *p* = 0.61 and *p* = 0.72, (Additional file 5: Table S3).

The distribution of serum VEGF variations during preoperative chemotherapy differed slightly between good and poor responders (median − 111 pg/mL versus −71 pg/mL, respectively; *p* = 0.06). Similarly, the proportion of poor responders was slightly lower among patients with a large decrease in VEGF, compared to others (first quartile of the distribution, Table 2). However, the relationship between the change in serum VEGF levels during preoperative treatment and the risk of a poor response was not monotonic, as illustrated by the odds ratio for a poor response according to the quartile of the distribution of serum VEGF variations. A non significant trend towards a relationship between VEGF variations and the histological response was also observed in the sensitivity analysis restricted to patients with high values at diagnosis (Additional file 6: Table S4).

**Table 2:** Serum-VEGF, serum-bFGF and urinary-bFGF variations, and risk of poor histological response or treatment failure

	Risk of poor histological response			Risk of treatment failure		
Variation between baseline and pre-surgery(T1-T0)	Poor Resp./ N^a^	Adjusted Odds Ratio (95%CI)^b^	*P* value	Event / N^c^	Adjusted Hazard Ratio (95%CI)^d^	*P* value
Serum VEGF (*N* = 165)			0.26			0.67
Q1: −1424 to −284	12 / 44	1 (ref)		16 / 44	1 (ref)	
Q2: −279 to --80	16 / 43	1.55 (0.57–4.2)		16 / 45	1.13 (0.52–2.5)	
Q3: −79 to − + 35	12 / 39	1.39 (0.46–4.2)		12 / 45	0.98 (0.41–2.3)	
Q4: − + 39 to +503	19 / 39	2.63 (0.97–7.1)		12 / 45	0.69 (0.29–1.7)	
Serum bFGF (*N* = 167)			0.85			0.39
Q1: −257 to −2.5	15 / 44	1 (ref)		14 / 45	1 (ref)	
Q2: −2.4 to +0.8	14 / 44	0.66 (0.21–2.0)		16 / 45	0.66 (0.22–2)	
Q3: +0.9 to +7.2	15 / 40	0.89 (0.3–2.7)		16 / 45	0.45 (0.15–1.3)	
Q4: +7.8 to +92	15 / 39	0.92 (0.3–2.9)		11 / 45	0.45 (0.14–1.4)	
Urinary bFGF (*N* = 73)			0.67			0.20
Q1: −14.4 to −2.1	5 / 18	1 (ref)		11 / 19	1(ref)	
Q2: −2.1 to +2.4	3 / 17	0.48 (0.08–3.0)		2 / 19	0.17 (0.03–0.89)	
Q3: +2.6 to +12.9	5 / 19	0.70 (0.13–3.9)		6 / 20	0.54 (0.15–1.9)	
Q4: +13.8 to +413	7 / 19	1.32 (0.30–5.7)		9 / 19	0.51 (0.18–1.5)	

^a^Poor Resp. / *N*: number of patients with poor histological response / number of evaluated patients

^b^Adjusted odds ratios and their 95% confidence intervals were estimated by multivariable logistic regression, including the biomarker level at diagnosis in quartiles. Results were similar when the model also included the treatment arm (with versus without zoledronate)

^c^Events / *N*: number of events in each subset / number of patients

^d^Hazard ratios of treatment failure and their 95% confidence intervals were estimated in Cox models controlling for the treatment group, initial stage and biomarker level at diagnosis in quartiles

**Fig. 2:**
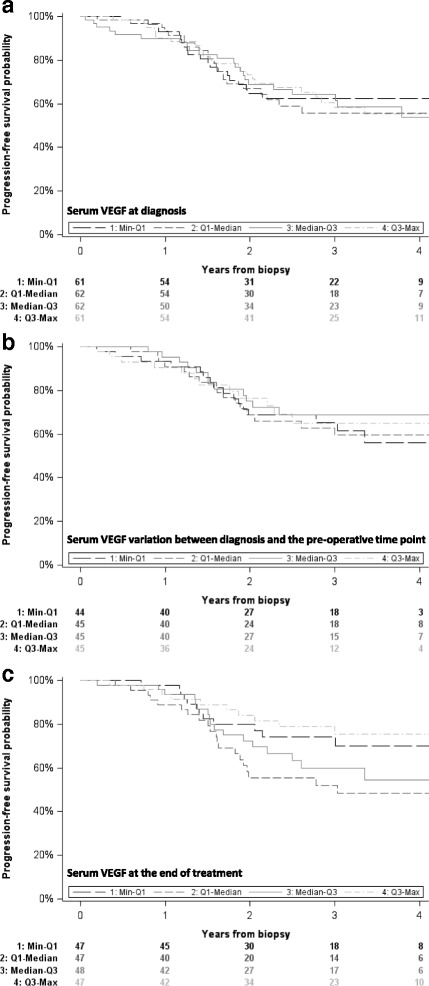
Progression-free survival according to VEGF levels and their variation. Panel A: serum VEGF at diagnosis, Panel B: serum VEGF variation between diagnosis and the pre-operative time point, Panel C: serum VEGF at the end of treatment

As illustrated by the progression-free survival curves (Fig. 2), we observed no relationship between the risk of treatment failure and A) the serum VEGF level at T0, B) the variation in serum VEGF levels between T0 and T1, or C) the serum VEGF level at T2. Multivariable models yielded similar conclusions, as shown in Table 2 for the variation between T0 and T1. Similar result was obtained for overall survival (*p* = 0.87).

We observed no trend between the studied outcomes and plasma VEGF levels at diagnosis (*p* = 0.85 for the histological response, *p* = 0.16 for the progression-free survival and 0.40 for the overall survival, details available upon request), change in serum bFGF, urinary bFGF (Table 2), plasma VEGF or plasma bFGF levels (Additional file 7: Table S5).

## Discussion

Angiogenic factors were detectable in biological fluids of all patients with newly diagnosed osteosarcoma included in this study, who had been prospectively recruited. Using published cutoffs [14, 18], most patients had high levels of VEGF and/or bFGF at diagnosis. However, it is difficult to compare the study population with control subjects used to establish reference values, because of the widespread distribution of the patient’s age; indeed, reference values are generally established in small series, separately in healthy young children for serum VEGF and bFGF and urinary bFGF [14], and in adults for serum VEGF and bFGF [18]. Variations in angiogenic factor levels may also be related to the fluid in which they are analysed. Most previous studies of bFGF and VEGF in blood have used serum samples but, because platelets contain VEGF, some authors have suggested that plasma may be more suitable for VEGF measurement [19]. As this controversy had still not been resolved at the time of this study [20], we measured VEGF in both serum and plasma. Serum and plasma values were related, but with a weak correlation coefficient.

Several authors have previously investigated angiogenic factor blood levels in patients with osteosarcoma with conflicting conclusions [21–25]. Significantly higher levels of VEGF were observed in bone sarcoma patients compared to healthy controls in three articles [23–25], whereas no significant difference was reported in the two others [21, 22]. However, each of these studies included less than 50 osteosarcoma patients. Using the same VEGF assay method, Rutkowski et al. found a significant association between serum levels at diagnosis and tumour size [25], which is consistent with our findings. This may reflect the need for new blood vessels formation associated with bone remodelling process for local tumour extension. Nevertheless, the reported impact of these high levels on patient outcomes is discordant. Some authors found a negative prognostic impact of high VEGF levels [23, 24, 26]. In our series, the largest so far conducted in this setting, VEGF levels had no significant impact on outcome, in agreement with Rutkowski et al. [25]. As in previous studies of bFGF [13, 25], we found no clinical value of this angiogenic factor in osteosarcoma. Here, as in the study by Rastogi et al. [23], serum VEGF levels fell significantly between baseline and surgery, with a slightly larger variation in patients who had a good histologic response. However, this variation was not associated with better progression-free survival.

The prognostic values of microvessel density and of angiogenic factor expression in osteosarcoma tissue sections were not evaluated in this study, but are also a matter of debate. Microvessel density and VEGF expression in tumours were found to be associated with a poor prognosis in small series of patients [5–8, 27]. Two meta-analyses suggest that VEGF expression is an effective prognostic biomarker in patients with osteosarcoma [28, 29]. In addition, a correlation has been found between VEGF expression after neoadjuvant chemotherapy and histological necrosis [30]. Using RT-PCR to detect VEGF isoforms in tumour samples of 30 non metastatic osteosarcomas, Lee et al. reported a significantly poorer prognosis among patients expressing the isoform VEGF-165 (detected by our technique) [4]. A prognostic impact of the change in VEGF expression in tumour specimens between diagnosis and post-chemotherapy resection was also found recently in a series of 61 Chinese patients, with significantly better survival among patients with a large reduction in VEGF expression after neoadjuvant chemotherapy as compared with those with a small reduction [31]. On the other hand, some other authors found no prognostic impact of microvessel density or VEGF or bFGF expression [10, 32–34]. In Kreuter’s study, microvessel density was actually associated with a better response to chemotherapy and with better survival, possibly owing in part to improved drug access to tumour cells [9].

The absence of significant association between blood VEGF levels and response to treatment and outcome in our study might be due to concealment of this effect by the intensity of our chemotherapy protocol; or alternatively to the need to evaluate a larger number of angiogenic factors (pro and anti-angiogenic) to understand the complexity of the process. Several authors have shown a correlation between vessel counts, VEGF expression in tumours and serum VEGF levels [21, 22], but blood levels of VEGF are unlikely to accurately reflect intra-tumour angiogenesis. Confounding factors such as surgical procedures or infections, which could not be taken into account in our analysis, could also have interfered with the results. Patients receiving intensive chemotherapy are at a high risk of febrile neutropenia and infection. Elevated blood levels of VEGF have been found during severe infections [35], and infectious adverse events could have minimized the fall in VEGF levels from baseline to the pre-surgical time point, thus explaining the slight increase in VEGF levels from the latter time point to the end of treatment.

Lastly, the possible impact of zoledronate on our results deserves discussion. Zoledronate has been shown to have an anti-angiogenic effect in a number of cancer models [36]. However, in our clinical trial the decrease in serum VEGF during pre-operative chemotherapy was similar regardless of zoledronate administration, making it unlikely that use of this drug influenced our results.

Despite the absence of prognostic impact of VEGF levels in our study, encouraging tumour responses obtained with anti-angiogenic agents in experimental osteosarcoma models, phase II clinical trials, and case reports [37–39] suggest that angiogenesis could play a major role in osteosarcoma progression. Further investigations of drugs targeting this pathway in osteosarcoma are ongoing.

## Conclusions

VEGF and bFGF blood levels are not associated with response to treatment or outcome. Our results are not in favour of monitoring blood and urinary angiogenic factors during conventional chemotherapy in patients with osteosarcoma.

## Additional files

Additional file 1: **Fig. S1.** Participant flow diagram (DOCX 30 kb)

Additional file 2: **Table S1.** Characteristics of included and excluded patients (DOCX 18 kb)

Additional file 3: **Table S2.** Distribution of plasma VEGF and bFGF levels at diagnosis, according to patient and tumour characteristics (DOCX 16 kb)

Additional file 4: **Fig. S2.** Correlation between the different biomarker levels at diagnosis (DOCX 23 kb)

Additional file 5: **Table S3.** Association between serum VEGF and bFGF and urinary bFGF levels at diagnosis and the risk of a poor histological response or treatment failure (multivariable analysis) (DOCX 16 kb)

Additional file 6: **Table S4.** Sensitivity analysis. Association between serum VEGF and bFGF and urine bFGF variations during pre-operative chemotherapy and the risk of a poor histological response or treatment failure among patients with high biomarker levels at diagnosis (multivariable analysis) (DOCX 16 kb)

Additional file 7: **Table S5.** Association between plasma VEGF and bFGF variations during pre-operative chemotherapy and the risk of a poor histological response or treatment failure (multivariable analysis) (DOCX 16 kb)

## Abbreviations

bFGF: basic fibroblast growth factor
HDMTX: High-dose methotrexate
OS: Overall survival
PFS: Progression-free survival
VEGF: Vascular endothelial growth factor
