# Supplementary figures and images for: Prognostic impact of blood and urinary angiogenic factor levels at diagnosis and during treatment in patients with osteosarcoma: a prospective study
Source: BMC Cancer. 2017 Jun 15;17:419. doi: 10.1186/s12885-017-3409-z (PMC5473001; doi:10.1186/s12885-017-3409-z)

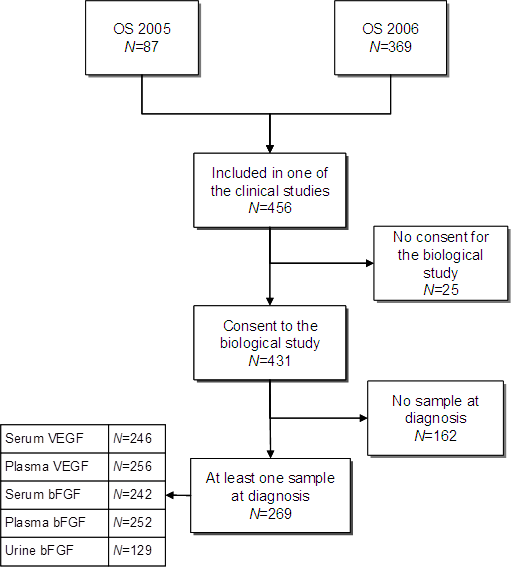


**Figure-S1: Participant flow diagram**

Supplement: Supplementary file 1 — Fig. S1. Participant flow diagram (DOCX 30 kb) [file 12885_2017_3409_MOESM1_ESM.docx]
