# Supplementary material for: Prognostic impact of blood and urinary angiogenic factor levels at diagnosis and during treatment in patients with osteosarcoma: a prospective study
Source: BMC Cancer. 2017 Jun 15;17:419. doi: 10.1186/s12885-017-3409-z (PMC5473001; doi:10.1186/s12885-017-3409-z)
Supplement: Supplementary file 2 — Table S1. Characteristics of included and excluded patients (DOCX 18 kb) [file 12885_2017_3409_MOESM2_ESM.docx]

**Table-S1: Characteristics of included and excluded patients**

| **Characteristics** | **No result at diagnosis** | **At least one result at diagnosis** | **Total** | ***P value*^1^** |
| --- | --- | --- | --- | --- |
|  | *N* (%) | *N* (%) | *N* |  |
| Overall | 187 | 269 | 456 |  |
| **Gender** |  |  |  | 0.46 |
| Male | 108 (58) | 146 (54) | 254 |  |
| Female | 79(42) | 123(46) | 202 |  |
| **Age** |  |  |  | 0.46 |
| <13 years | 50 (27) | 80 (30) | 130 |  |
| 13-18 years | 77 (41) | 117 (43) | 194 |  |
| >18 years | 60 (32) | 72 (27) | 132 |  |
| **Tumour site** |  |  |  | 0.55 |
| Femur diaphysis | 9 (5) | 20 (8) | 29 |  |
| Femur inferior extremity | 62 (37) | 109 (41) | 171 |  |
| Femur superior extremity | 9 (5) | 10 (4) | 19 |  |
| Tibia superior extremity | 30 (18) | 43 (16) | 73 |  |
| Humerus superior extremity | 10 (6) | 22 (8) | 32 |  |
| Other | 48 (29) | 61 (23) | 109 |  |
| Missing data | 19 | 4 | 23 |  |
| **Tumour size** |  |  |  | 0.57 |
| <10 cm | 76 (49) | 116 (46) | 192 |  |
| ≥10 cm | 80(51) | 137 (54) | 217 |  |
| Missing data | 31 | 16 | 47 |  |
| **Initial stage** |  |  |  | 0.41 |
| Localized | 105 (62) | 181 (68) | 286 |  |
| Doubtful lesions | 31 (18) | 44 (17) | 75 |  |
| Metastases | 33 (20) | 41 (15) | 74 |  |
| Missing data | 18 | 3 | 21 |  |
| **Histologic subtype** |  |  |  | 0.18**^2^** |
| Osteoblastic | 95 (59) | 170 (66) | 265 |  |
| Fibroblastic | 7 (4) | 12 (5) | 19 |  |
| Chondroblastic | 25 (15) | 43 (17) | 68 |  |
| Telangiectasic | 5 (3) | 7 (3) | 12 |  |
| Other | 30 (19) | 26 (10) | 56 |  |
| - Giant cells | 1 | 2 |  |  |
| - Juxtacortical low grade | 1 | 1 |  |  |
| - Surface high grade | 5 | 1 |  |  |
| - Well-differentiated | 2 | 0 |  |  |
| - Secondary | 0 | 1 |  |  |
| - Miscellaneous | 21 | 21 |  |  |
| Missing data | 25 | 11 | 36 |  |
| **Alkaline phosphatase** |  |  |  | 0.31 |
| < 1.25 x ULN | 66 (61) | 149 (66) | 215 |  |
| > 1.25 x ULN | 43 (39) | 76 (34) | 119 |  |
| Missing data | 78 | 44 | 122 |  |
| **Treatment arm (OS2006 trial)** |  |  |  | 0.60 |
| Without zoledronate | 91 (71) | 165 (68) | 256 |  |
| With zoledronate | 37 (29) | 76 (32) | 113 |  |

**^1^** The *P values* were computed without considering missing values.

**^2^** The *P value* was computed after pooling “Giant cells”, “Juxtacortical or parosteal low-grade”, “Surface high-grade”, “Well-differentiated”, “Secondary” and “Miscellaneous”.
