# Supplementary material for: Prognostic impact of blood and urinary angiogenic factor levels at diagnosis and during treatment in patients with osteosarcoma: a prospective study
Source: BMC Cancer. 2017 Jun 15;17:419. doi: 10.1186/s12885-017-3409-z (PMC5473001; doi:10.1186/s12885-017-3409-z)
Supplement: Supplementary file 3 — Table S2. Distribution of plasma VEGF and bFGF levels at diagnosis, according to patient and tumour characteristics (DOCX 16 kb) [file 12885_2017_3409_MOESM3_ESM.docx]

**Table-S2: Distribution of plasma VEGF and bFGF levels at diagnosis, according to patient and tumour characteristics**

|  | **Plasma VEGF (pg/mL)** | | | **Plasma bFGF (pg/mL)** | | |
| --- | --- | --- | --- | --- | --- | --- |
|  | *N* | Median [Q1-Q3] ^1^ | *P value*^2^ | *N* | Median [Q1;Q3]^1^ | *P value*^2^ |
| **All** | 256 | 84 [55-164] |  | 252 | 4 [3-12] |  |
| **Gender** |  |  | 0.95 |  |  | 0.46 |
| Male | 136 | 85 [57-149] |  | 135 | 4 [3-13] |  |
| Female | 120 | 81 [52-191] |  | 117 | 3 [3-12] |  |
| **Age** |  |  | 0.66 |  |  | 0.06 |
| <13 years | 75 | 95 [55-173] |  | 73 | 5 [3-14] |  |
| 13-18 years | 110 | 85 [57-190] |  | 110 | 4 [3-15] |  |
| >18 years | 71 | 81 [53-139] |  | 69 | 3 [3-8] |  |
| **tumour size** |  |  | 0.02 |  |  | 0.29 |
| <10 cm | 109 | 74 [49-139] |  | 108 | 5 [3-13] |  |
| ≥10 cm | 131 | 101 [59-178] |  | 129 | 3 [3-12] |  |
| **Initial stage** |  |  | 0.70 |  |  | 0.17 |
| Localized | 171 | 85 [55-176] |  | 169 | 4 [3-13] |  |
| Doubtful lesions | 42 | 88 [55-125] |  | 42 | 7 [3-12] |  |
| Metastases | 40 | 66 [48-160] |  | 38 | 3 [3-9] |  |
| **Histologic subtype** |  |  | 0.60 |  |  | 0.85 |
| Osteoblastic | 161 | 99 [55-192] |  | 160 | 4 [3-14] |  |
| Fibroblastic | 12 | 75 [56-117] |  | 12 | 6 [3-14] |  |
| Chondroblastic | 41 | 80 [58-136] |  | 39 | 3 [3-10] |  |
| Telangiectasic | 7 | 75 [44-258] |  | 7 | 3 [3-19] |  |
| Other | 24 | 83 [53-126] |  | 23 | 6 [3-12] |  |
| **Alkaline phosphatase** |  |  | 0.12 |  |  | 0.06 |
| < 1.25 x ULN | 143 | 81 [52-149] |  | 141 | 5 [3-13] |  |
| > 1.25 x ULN | 73 | 112 [60-164] |  | 72 | 3 [3-10] |  |
| **Histological response** |  |  | 0.08 |  |  | 0.08 |
| Good | 150 | 87 (57-190] |  | 148 | 5 [3-18] |  |
| Poor | 75 | 90 [57-147] |  | 74 | 3 [3-10] |  |

^1^ [Q1-Q3]: inter-quartile range.

^2^ *P value* of the Kruskal-Wallis test comparing the distributions between the different subsets.
