# Supplementary material for: Prognostic impact of blood and urinary angiogenic factor levels at diagnosis and during treatment in patients with osteosarcoma: a prospective study
Source: BMC Cancer. 2017 Jun 15;17:419. doi: 10.1186/s12885-017-3409-z (PMC5473001; doi:10.1186/s12885-017-3409-z)
Supplement: Supplementary file 4 — Fig. S2. Correlation between the different biomarker levels at diagnosis (DOCX 23 kb) [file 12885_2017_3409_MOESM4_ESM.docx]

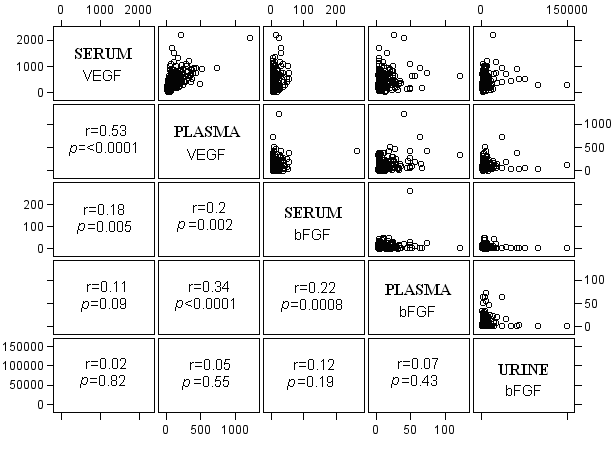


Figure-S2: Correlation between the different biomarkers at diagnosis

For each pair of biomarkers,

- - r: Spearman correlation coefficient
  - *p*: P value in a non-parametric test of the null hypothesis, r=0
