# Supplementary material for: Prognostic impact of blood and urinary angiogenic factor levels at diagnosis and during treatment in patients with osteosarcoma: a prospective study
Source: BMC Cancer. 2017 Jun 15;17:419. doi: 10.1186/s12885-017-3409-z (PMC5473001; doi:10.1186/s12885-017-3409-z)
Supplement: Supplementary file 5 — Table S3. Association between serum VEGF and bFGF and urinary bFGF levels at diagnosis and the risk of a poor histological response or treatment failure (multivariable analysis) (DOCX 16 kb) [file 12885_2017_3409_MOESM5_ESM.docx]

**Table-S3: Association between serum VEGF and bFGF and urine bFGF levels at diagnosis and the risk of a poor histological response or treatment failure (multivariable analysis)**

|  | **Risk of poor histological response** | | | **Risk of treatment failure** | | |
| --- | --- | --- | --- | --- | --- | --- |
| **Baseline** | **Poor Resp. */ N***^1^ | **Adjusted Odds Ratio (95%CI)**^2^ | ***P value*** | **Event / *N***^3^ | **Adjusted Hazard Ratio (95%CI)**^4^ | ***P value*** |
| **Serum VEGF (N=214)** |  |  | 0.44 |  |  | 0.95 |
| Q1: 29 to 271 | 15 / 48 | 1 (ref) |  | 20 / 61 | 1 (ref) |  |
| Q2: 274 to 428 | 23 / 58 | 1.45 (0.65-3.24) |  | 22 / 62 | 0.99 (0.53-1.84) |  |
| Q3: 429 to 685 | 21 / 50 | 1.59 (0.70-3.65) |  | 22 / 62 | 0.90 (0.49-1.68) |  |
| Q4: 686 to 2219 | 17 / 58 | 0.91 (0.40-2.10) |  | 26 / 61 | 1.08 (0.60-1.96) |  |
| **Serum bFGF (N=211)** |  |  | 0.61 |  |  | 0.44 |
| Q1: 1.9 to 3 | 33 / 89 | 1 (ref) |  | 47 / 101 | 1 (ref) |  |
| Q2: 3.1 to 4.2 | 7 / 17 | 1.19 (0.41-3.42) |  | 6 / 20 | 0.79 (0.33-1.89) |  |
| Q3: 4.4 to 12.5 | 20 / 54 | 0.99 (0.50-2.01) |  | 16 / 61 | 0.64 (0.36-1.15) |  |
| Q4: 12.6 to 265 | 14 / 51 | 0.64 (0.30-1.36) |  | 19 / 60 | 0.75 (0.43-1.29) |  |
| **Urine bFGF (N=116)** |  |  | 0.72 |  |  | 0.76 |
| Q1: 0.7 to 2.6 | 10 / 29 | 1 (ref) |  | 9 / 32 | 1(ref) |  |
| Q2: 2.7 to 5.1 | 8 / 32 | 0.63 (0.21-1.92) |  | 9 / 32 | 0.80 (0.31-2.09) |  |
| Q3: 5.2 to 10.4 | 6 / 28 | 0.52 (0.16-1.69) |  | 13 / 33 | 1.11 (0.46-2.68) |  |
| Q4: 10.8 to 15 | 7 / 27 | 0.67 (0.21-2.10) |  | 15 / 32 | 1.27 (0.54-2.98) |  |

**^1^**Poor Resp. / *N*: number of patients with a poor histological response / number of evaluated patients.

**^2^**Adjusted Odds Ratios and their 95% confidence intervals were estimated by univariate logistic regression. Results were similar when the model also included the treatment arm (with versus without zoledronate).

**^3^**Events / *N*: number of events in each subset / number of patients.

**^4^**Hazard ratios of treatment failure (progression, relapse or death) and their 95% confidence intervals were estimated in Cox models controlling for the treatment group, and initial stage.
