# Supplementary material for: Prognostic impact of blood and urinary angiogenic factor levels at diagnosis and during treatment in patients with osteosarcoma: a prospective study
Source: BMC Cancer. 2017 Jun 15;17:419. doi: 10.1186/s12885-017-3409-z (PMC5473001; doi:10.1186/s12885-017-3409-z)
Supplement: Supplementary file 6 — Table S4. Sensitivity analysis. Association between serum VEGF and bFGF and urine bFGF variations during pre-operative chemotherapy and the risk of a poor histological response or treatment failure among patients with high biomarker levels at diagnosis (multivariable analysis) (DOCX 16 kb) [file 12885_2017_3409_MOESM6_ESM.docx]

**Table-S4: Sensitivity analysis. Association between serum VEGF and bFGF and urine bFGF variations during pre-operative chemotherapy and the risk of a poor histological response or treatment failure among patients with high biomarker levels at diagnosis (multivariable analysis)**

|  | **Risk of poor histological response** | | | **Risk of treatment failure** | | |
| --- | --- | --- | --- | --- | --- | --- |
| **Variation between baseline and pre-surgery (T1-T0)** | **Poor Resp. */ N***^1^ | **Adjusted Odds Ratio (95%CI)**^2^ | ***P value*** | **Event / *N***^3^ | **Adjusted Hazard Ratio (95%CI)**^5^ | ***P value*** |
| **Serum VEGF** ^4^ **(N=100)** |  |  | 0.09 |  |  | 0.71 |
| Q1: -1424 to -385 | 4 / 25 | 1 (ref) |  | 10 / 25 | 1 (ref) |  |
| Q2: -377 to -216 | 11 / 26 | 3.99 (0.74-22) |  | 8 / 26 | 1.46 (0.37-5.72) |  |
| Q3: -212 to -21 | 11 / 25 | 4.34 (0.91-21) |  | 7 / 26 | 0.87 (0.27-2.85) |  |
| Q4 : -8 to +492 | 13 / 24 | 7.74 (1.55-39) |  | 8 / 26 | 1.55 (0.49-4.89) |  |
| **Serum bFGF**^4^ **(N=87)** |  |  | 0.13 |  |  | 0.22 |
| Q1: -257 to -8.8 | 10 / 22 | 1 (ref) |  | 8 / 23 | 1 (ref) |  |
| Q2: -8.1 to -2.5 | 6 / 23 | 0.14 (0.02-0.82) |  | 7 / 23 | 1.19 (0.28-4.97) |  |
| Q3: -2.4 to +2.6 | 8 / 24 | 0.19 (0.03-1.18) |  | 5 / 24 | 0.25 (0.04-1.49) |  |
| Q4: +3 to +57 | 9 / 18 | 0.39 (0.07-2.39) |  | 3 / 23 | 0.41 (0.06-2.61) |  |
| **Urine bFGF**^4^ **(N=70)** |  |  | 0.50 |  |  | 0.10 |
| Q1: -14.4 to -2.4 | 5 / 17 | 1 (ref) |  | 11 / 18 | 1(ref) |  |
| Q2: -2.1 to +2.3 | 3 / 17 | 0.30 (0.04-2.12) |  | 2 / 19 | 0.12 (0.02-0.66) |  |
| Q3: +2.4 to +1.3 | 5 / 18 | 0.55 (0.10-3.07) |  | 6 / 19 | 0.46 (0.14-1.57) |  |
| Q4: +1.4 to +4.1 | 7 / 18 | 1.04 (0.22-4.92) |  | 8 / 18 | 0.35 (0.10-1.17) |  |

**^1^**Poor Resp. / *N*: number of patients with a poor histological response / number of evaluated patients.

**^2^**Adjusted Odds Ratios and their 95% confidence intervals were estimated by multivariable logistic regression, including the biomarker level at diagnosis in quartiles. Results were similar when the model also included the treatment arm (with versus without zoledronate).

^3^Events / *N*: number of events in each subset / number of patients.

^4^Adjusted Odds Ratios and their 95% confidence intervals were estimated by multivariable logistic regression, including the biomarker level at diagnosis.

**^5^**Hazard ratios of treatment failure (progression, relapse or death) and their 95% confidence intervals were estimated in Cox models controlling for the treatment group, initial stage and the biomarker level at diagnosis in quartiles.
